# Comparative efficacy and safety of different combinations of three CDK4/6 inhibitors with endocrine therapies in HR+/HER-2 − metastatic or advanced breast cancer patients: a network meta-analysis

**DOI:** 10.1186/s12885-023-11322-2

**Published:** 2023-08-31

**Authors:** Yiyuan Liu, Jinyao Wu, Zeqi Ji, Lingzhi Chen, Juan Zou, Jiehua Zheng, Weixun Lin, Jiehui Cai, Yaokun Chen, Daitian Zheng, Yexi Chen, Zhiyang Li

**Affiliations:** Department of Thyroid, Breast and Hernia Surgery, The Second Affiliated Hospital of Shantou, University Medical College, Shantou, Guangdong China

**Keywords:** Breast cancer, Abemaciclib, Ribociclib, Palbociclib, Network meta-analysis

## Abstract

**Background:**

This network meta-analysis aimed to assess the comparative efficacy and safety of combinations involving three cyclin-dependent kinase 4/6 (CDK4/6) inhibitors and endocrine therapies (ETs) in patients with metastatic or advanced breast cancer (BC) who are hormone receptor-positive (HR+) and human epidermal growth factor receptor 2-negative (HER2-).

**Methods:**

We initially identified relevant studies from previous meta-analyses and then conducted a comprehensive search of PubMed, Embase, and the Cochrane Central Register of Controlled Trials (CENTRAL) databases to locate additional studies published between February 2020 and September 2021. Essential data were extracted, and a network meta-analysis was performed using R 4.1.1 software with a random-effects model. Furthermore, we assigned rankings to all available treatment combinations by calculating their cumulative probability.

**Results:**

Data analysis included ten reports from nine studies. Pooled results demonstrated that each treatment combination significantly reduced the hazard risk of progression-free survival (PFS) compared to treatment with an aromatase inhibitor (AI) or fulvestrant alone. However, there were no differences observed in PFS or overall survival (OS) among the different treatment combinations. Additionally, patients receiving palbociclib plus AI and abemaciclib plus AI or fulvestrant experienced more severe adverse events (AEs), with hazard ratios (HRs) of 10.83 (95% confidence interval [CI] = 2.3 to 52.51) and 4.8 (95%CI = 1.41 to 16.21), respectively. The HR for ribociclib plus AI was 9.45 (95%CI = 2.02 to 43.61), and the HR for palbociclib plus fulvestrant was 6.33 (95%CI = 1.03 to 39.86). Based on the ranking probabilities, palbociclib plus fulvestrant had the highest probability of achieving superior PFS (37.65%), followed by abemaciclib plus fulvestrant (28.76%). For OS, ribociclib plus fulvestrant ranked first (34.11%), with abemaciclib plus fulvestrant in second place (25.75%). In terms of safety, palbociclib plus AI (53.98%) or fulvestrant (51.37%) had the highest probabilities of being associated with adverse events.

**Conclusions:**

Abemaciclib plus fulvestrant or ribociclib plus AI appear to be effective and relatively safe for the treatment of HR+/HER2- metastatic or advanced BC patients. However, given the reliance on limited evidence, our findings require further validation through additional studies.

**Supplementary Information:**

The online version contains supplementary material available at 10.1186/s12885-023-11322-2.

## Background

According to the Global Cancer Statistics 2020 report, breast cancer (BC) has the highest incidence worldwide among all cancer types and is the leading cause of cancer-related death in women [1]. Studies have indicated that approximately 30–40% of early-stage BC patients eventually progress to advanced BC [2], with a small proportion of patients already diagnosed with distant metastases [3]. A recent study by Shi et al. highlighted COL11A1 as a potential novel biomarker for BC, as it was found to be highly expressed in BC samples and associated with poor prognosis. This suggests that COL11A1 could be a potential therapeutic target in BC. However, it is important to note that BC exhibits significant heterogeneity [4], leading to the identification of four distinct molecular subtypes based on the presence of different biomarkers [5]. Among these subtypes, hormone receptor-positive (HR+) and human epidermal growth factor receptor 2-negative (HER2-) BC are the most common, accounting for 60–65% of patients with metastatic or advanced BC [3, 6, 7]. Traditionally, endocrine therapy, including aromatase inhibitors (AI) or fulvestrant, is recommended as first-line treatment for HR+/HER2- BC patients, unless there is a visceral crisis or life-threatening situation [8, 9]. However, some patients either fail to respond to initial therapy due to primary resistance [10] or experience disease progression during treatment due to acquired resistance [11]. Cyclin-dependent kinase 4/6 (CDK4/6) plays a crucial role in cell cycle regulation [12] and has been closely associated with endocrine therapy resistance [13]. Notably, the US Food and Drug Administration (FDA) has approved three CDK4/6 inhibitors, namely palbociclib, ribociclib, and abemaciclib, for the treatment of HR+/HER2- BC patients [14]. Currently, there is increasing interest among researchers and practitioners in exploring the efficacy and safety of combining different CDK4/6 inhibitors with endocrine therapies [15]. Several pairwise [16–21] and network meta-analyses [22–24] have confirmed the efficacy and safety of CDK4/6 inhibitors in combination with endocrine therapy for the treatment of metastatic or advanced BC patients with HR+/HER2-. However, definitive investigations of the comparative efficacy and safety of different treatment combinations involving CDK4/6 inhibitors (abemaciclib, palbociclib, and ribociclib) and endocrine therapies (AI and fulvestrant) in clinical trials and previously published meta-analyses are lacking. It is worth noting that network meta-analysis allows for the summary and comparison of the efficacy and safety of different treatment combinations, facilitating their ranking based on cumulative probability [25]. Therefore, we conducted this network meta-analysis to investigate the comparative efficacy and safety of various combinations of three CDK4/6 inhibitors (abemaciclib, palbociclib, and ribociclib) and two endocrine therapies (AI and fulvestrant) in patients with HR+/HER2- metastatic or advanced BC, with the aim of providing evidence-based recommendations for clinical decision-making.

## Methods

### Study design

This network meta-analysis adhered strictly to the guidelines of the Preferred Reporting Items for Systematic Reviews and Meta-Analysis (PRISMA) for network meta-analysis (PRISMA-NMA) [26, 27]. We also followed the methodological framework recommended by the Cochrane Handbook for Reviewers of Systematic Reviews [28].

### Search Strategy

We initially identified systematic reviews and meta-analyses related to the topic in PubMed (Medline) using MeSH terms and corresponding synonyms: breast cancer, palbociclib, ribociclib, abemaciclib, and random. Subsequently, two independent authors (Yiyuan Liu and Jinyao Wu) conducted searches in PubMed, Embase (Ovid), and the Cochrane Central Register for Controlled Trials (CENTRAL) to retrieve additional studies published between February 2020 and September 2021, as the previous meta-analysis had screened studies published before 2020 [18]. The complete search strategy is summarized in Supplementary Table 1. Moreover, we manually reviewed the reference lists of eligible studies and related meta-analyses to identify additional studies. Literature retrieval was performed independently by two authors, with any discrepancies resolved through discussion with the senior author.

### Inclusion criteria

To be eligible for inclusion, studies needed to meet the following criteria: (a) patients must have had a definitive diagnosis of HR+/HER2- metastatic or advanced BC, (b) therapeutic regimes must have included various combinations of three CDK4/6 inhibitors (abemaciclib, palbociclib, ribociclib) and two endocrine therapies (AI and fulvestrant), (c) at least one of the following outcomes must have been reported: progression-free survival (PFS), overall survival (OS), and severe treatment-related adverse events (AEs), and (d) the studies must have been full-text randomized controlled trials (RCTs).

#### Exclusion criteria

Studies were excluded if they met at least one of the following criteria: (a) ineligible study designs such as phase I trials, observational studies, animal studies, commentaries, and subgroup analyses; (b) redundant reports by the same group based on the same dataset; and (c) studies published in languages other than English.

#### Study selection

Study selection was performed using EndNote X9 software. Duplicate studies were automatically excluded by the software. Next, the title and abstract of each study were screened to evaluate eligibility. Subsequently, the full text of each potentially eligible study was assessed. Finally, we included all RCTs that compared the efficacy and safety of one combination regimen with others for HR+/HER2- metastatic or advanced BC patients. Eligibility was determined independently by two authors, with any disagreements resolved through discussion with a third senior author.

### Data extraction

Data extraction was performed independently by two authors. The following data were extracted from each eligible study: first author’s name, study identifier, clinical trial identifier, publication year, study phase, sample size, median age, median follow-up duration, clinical characteristics of patients, details of therapeutic regimens, and outcomes of interest.

#### Definition of outcomes

In this study, we defined progression-free survival (PFS) as the time from randomization to the first occurrence of radiographic or clinical progression or death, and overall survival (OS) as the time from randomization to death. Severe treatment-related adverse events (AEs) were defined as events with a grade ≥ 3.

### Methodological Quality Assessment

The methodological quality of each individual study was independently assessed by two authors using the Cochrane Collaboration risk of bias assessment tool [29]. Six aspects were evaluated: randomization, allocation concealment, blinding methods, attrition rate, outcome reporting, and other sources of bias. Each aspect was labeled as low, unclear, or high risk based on the quality evaluation results. Quality assessment was performed by two authors, with any disagreements resolved through discussion with a third senior author. RevMan version 5.4 (Review Manager, the Cochrane Collaboration, 2020) was used to visually represent the risk of bias summary graphically.

### Statistical analysis

Traditional pairwise meta-analysis based on the random-effects model was conducted using RevMan software. Bayesian network meta-analysis was performed using R software (V.4.1.1) and the “gemtc” package [30–32] through Markov Chain Monte Carlo (MCMC) simulation. Hazard ratios (HRs) with 95% credible intervals (CI) were used to express PFS and OS for each study, while odds ratios (ORs) with 95% CI were used to express the risk of severe AEs. The random-effects model was selected for this network meta-analysis to generate relatively conservative estimates, as this model accounts for heterogeneity across trials within individual comparisons [33]. MCMC simulation was conducted with 20,000 burn-in iterations followed by 50,000 iterations and 4 Markov chains [34]. The convergence of the models was qualitatively assessed using Gelman-Rubin-Brooks, trace, and density plots [35] and quantitatively assessed using the potential scale reduced factor (PSRF) [36, 37]. Treatment combinations were ranked based on their probabilities using the “gemtc” package [38]. The global heterogeneity of the model was assessed using the heterogeneity index (I^2^) through the “mtc.anohe” command [39]. Since only indirect evidence was available for all comparisons, the split-node method was not employed to investigate inconsistency between direct and indirect estimates [31, 40]. Publication bias was not assessed due to the insufficient number of eligible studies (< 10 studies) [33].

## Results

### Included studies

**Fig. 1:**
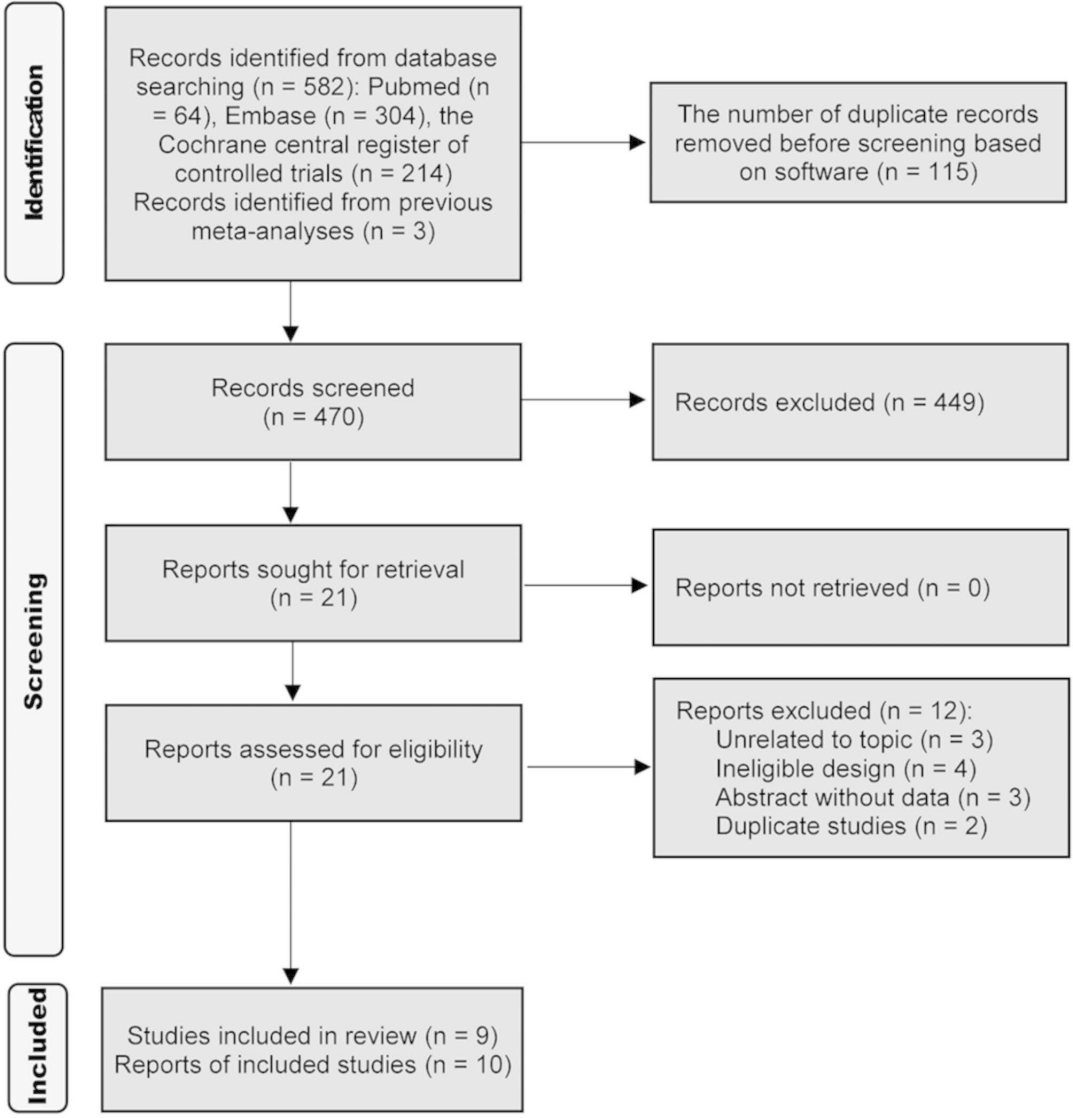
PRISMA flowchart of the study retrieval and selection process for this network meta-analysis

We identified 64, 304, and 241 potentially eligible studies from PubMed, Embase, and CENTRAL, respectively. After removing 115 duplicate studies using EndNote, we sequentially evaluated the titles, abstracts, and full texts. Ultimately, we deemed 6 studies eligible after excluding 461 ineligible studies, in addition to the 3 eligible studies identified from previous meta-analyses. Therefore, this network meta-analysis included a total of 9 eligible studies [41–49] comprising 10 reports. The study retrieval and selection process is presented in Fig. 1. Furthermore, to compare all available combinations for progression-free survival (PFS) and overall survival (OS), we included the results of a previous meta-analysis that examined the comparative efficacy and safety of fulvestrant and AI in advanced breast cancer [50] in this network meta-analysis.

### Baseline characteristics of included studies

**Fig. 2:**
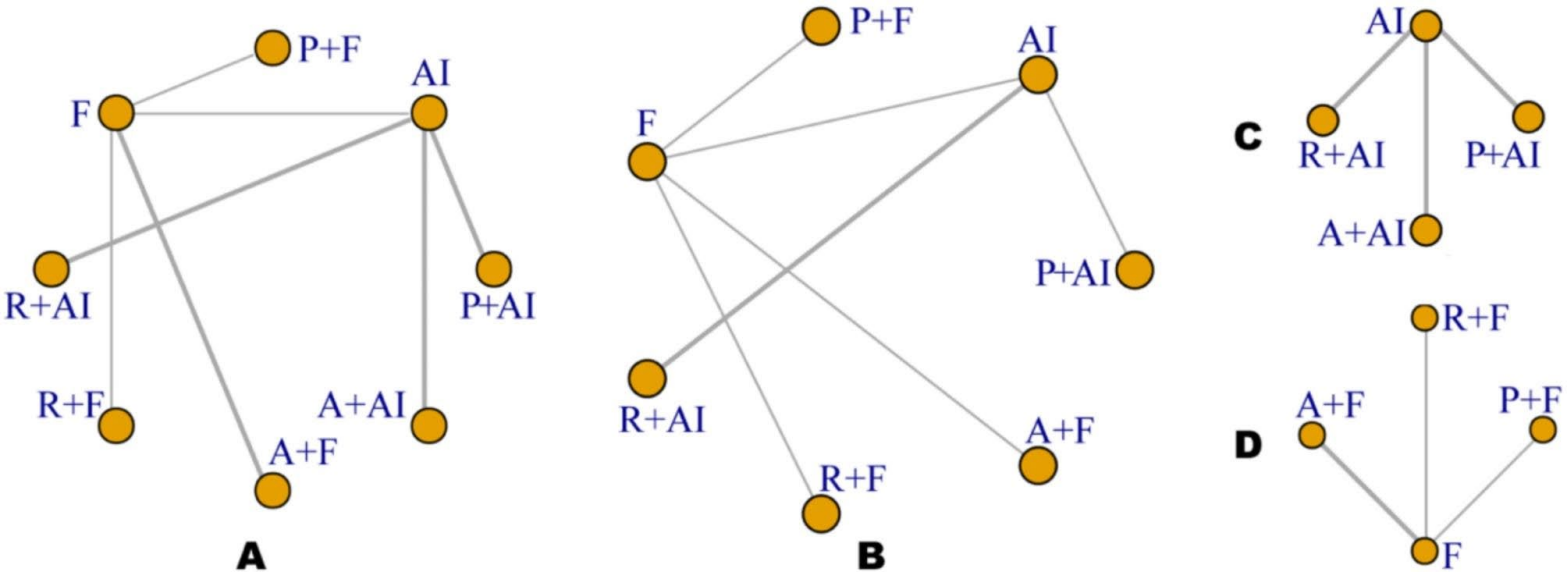
Network of the comparisons for the Bayesian network meta-analysis. As shown in the figure, the thickness of the lines is proportional to the number of comparisons, and the diameter of the circles is proportional to the number of treatments included in the meta-analysis. Network of PFS (A), OS (B), and severe AEs based on different endocrine therapies including AI (C) and fulvestrant (D)

Among the 9 studies included in this analysis, 2 studies [45, 46] were phase II design, while the remaining 7 studies [41–44, 47–49] were phase III designs. All of these studies provided data on the HR for PFS and the incidence of severe AEs, with an a total sample size of 5043 and 5022, respectively. Additionally, Six studies reported the HR for OS, involving a combined sample size of 3421. The evidence plots for PFS, OS, and severe AEs were presented in Fig. 2. In regard to severe AEs, we presented separate evidence plots based on different endocrine therapies, as there were no eligible studies exploring the connection between AI and fulvestrant. The baseline characteristics of the included studies are summarized in Supplementary Tables 2, and the numerical results for each outcome in each study can be found in Table 1.

**Table 1:** Reported HR for PFS and OS and severe AEs rate of each eligible study

Study	Study	Control	HR for PFS	HR for OS	Severe AEs rate, %
PALOMA-1	P + AI	AI	0.488 (0.319, 0.748)	0.9897 (0.623, 1.294)	75.90 vs. 20.78
PALOMA-2	P + AI	AI	0.563 (0.461, 0.687)	n.r.	79.28 vs. 28.38
PALOMA-3	P + F	F	0.50 (0.40, 0.62)	0.81 (0.64, 1.03)	72.33 vs. 21.84
MONALEESA-2	R + AI	AI	0.568 (0.457, 0.704)	0.746 (0.517, 1.078)	82.04 vs. 32.73
MONALEESA-3	R + F	F	0.593 (0.480, 0.732)	0.73 (0.59, 0.90)	28.51 vs. 16.53
MONALEESA-7	R + AI	AI	0.55 (0.44, 0.69)	0.71 (0.54, 0.95)	82.09 vs. 29.67
MONARCH-2	A + F	F	0.553 (0.449, 0.681)	0.757 (0.606, 0.945)	65.99 vs. 26.91
MONARCH-3	A + AI	AI	0.540 (0.418, 0.698)	n.r.	58.41 vs. 24.84
MONARCH plus (cohort A)	A + AI	AI	0.499 (0.346, 0.719)	n.r.	59.02 vs. 23.23
MONARCH plus (cohort B)	A + F	F	0.376 (0.240, 0.588)	n.r.	51.92 vs. 15.09

P, palbociclib; R, ribociclib; A, abemaciclib; AI, aromatase inhibitor; F, fulvestrant; AEs, adverse events; HR, hazard ratio; PFS, progression-free survival; OS, overall survival; n.r., not reported

### Quality Assessment of included studies

**Fig. 3:**
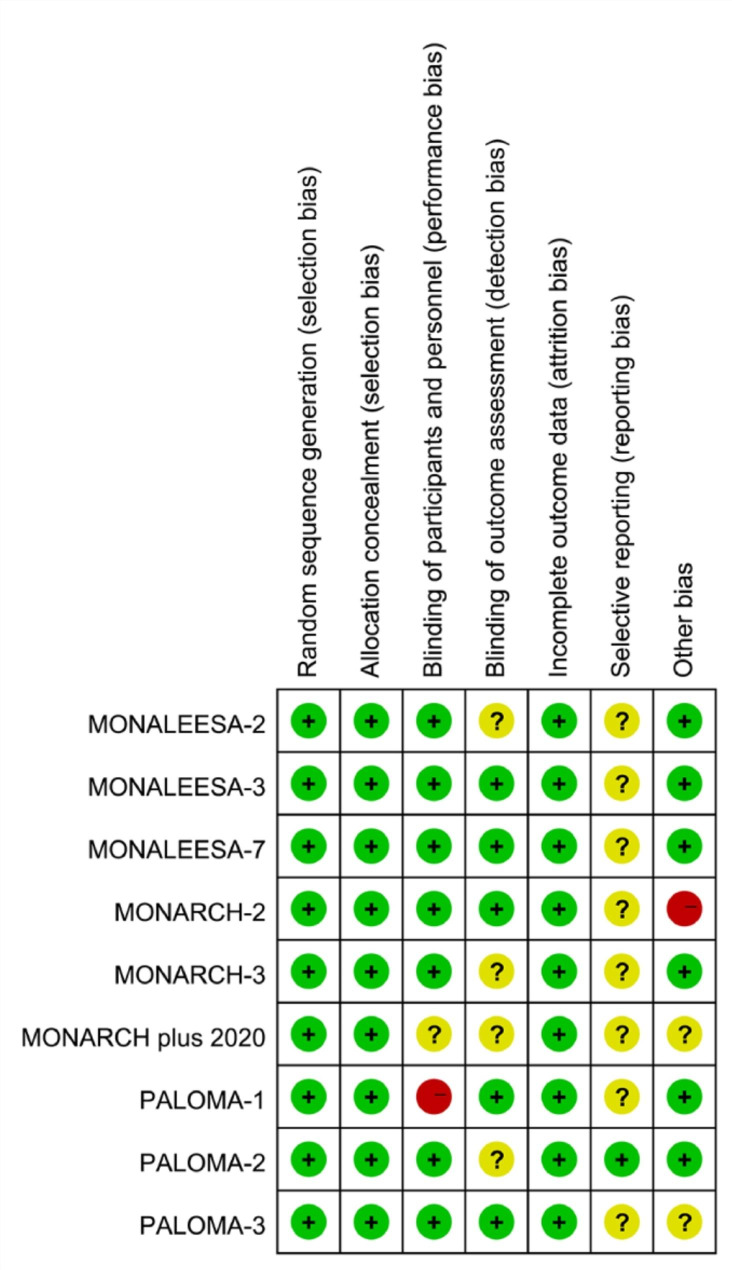
Risk of bias summary: reviewers’ judgments of each risk of bias item for each eligible study

Among the identified studies [41–49], all were considered to have a low risk of selection bias. Most studies effectively minimized performance and detection bias through double-blind designs, except for one study that used an open-label design [46]. Since attrition bias did not affect our estimates significantly, we categorized all studies as low risk in this domain. Regarding outcome reporting and other biases, most studies were either unclear or at low risk. A summary of the individual study-level assessment can be found in Fig. 3.

### Meta-analysis of progression-free survival

**Fig. 4:**
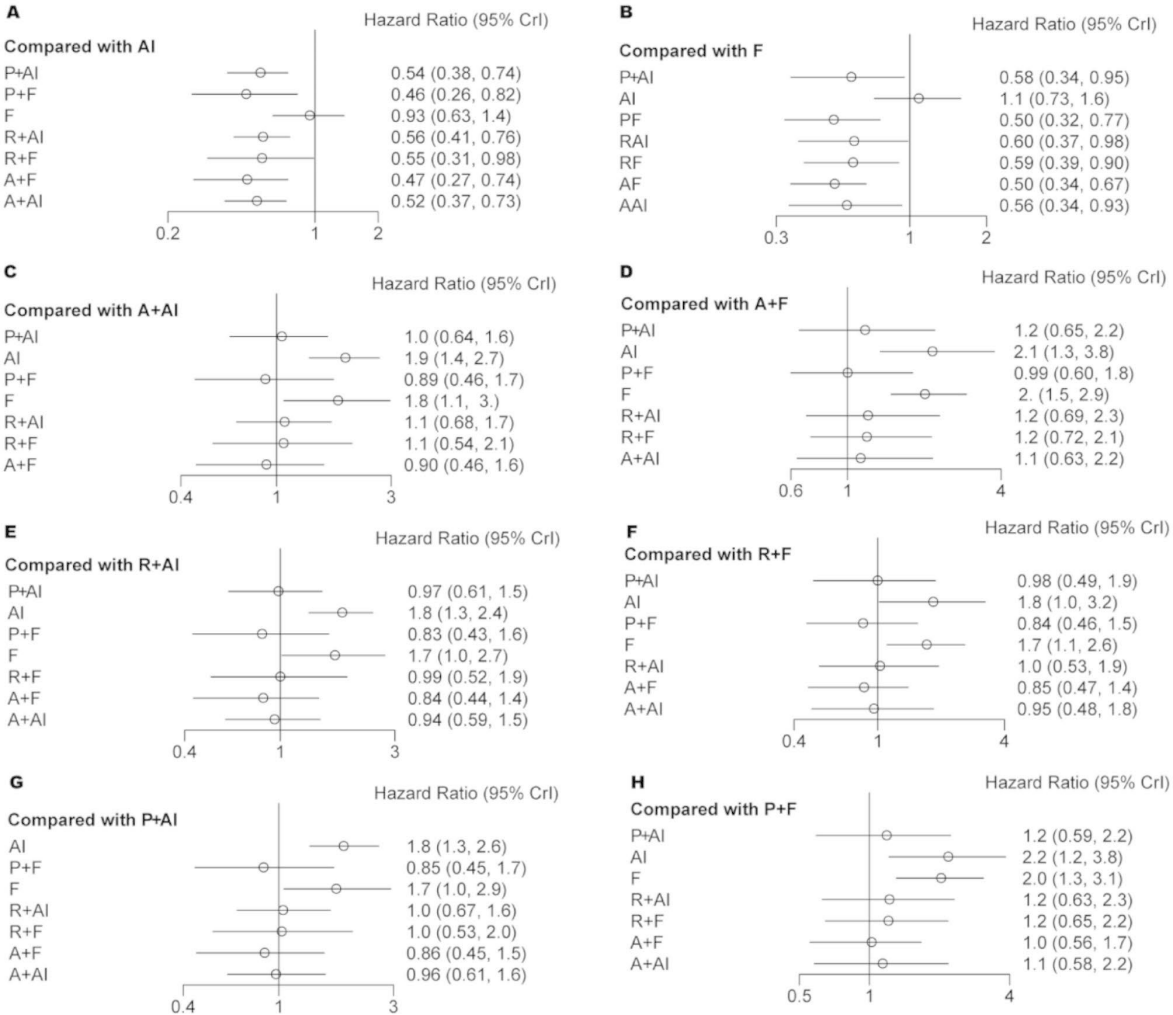
Forest plot of the hazard ratios for PFS based on different pairwise comparisons

All 9 eligible studies [41–49] involving 5043 patients reported hazard ratios (HR) for PFS. The pairwise meta-analysis results indicated a reduced hazard risk of PFS for each treatment combination (Supplementary Fig. 1). This finding was further supported by the network meta-analysis (Fig. 4). However, when comparing the available treatment combinations of three CDK4/6 inhibitors and various endocrine therapies, no statistical differences were observed for PFS (Fig. 4).

### Meta-analysis of overall survival

**Fig. 5:**
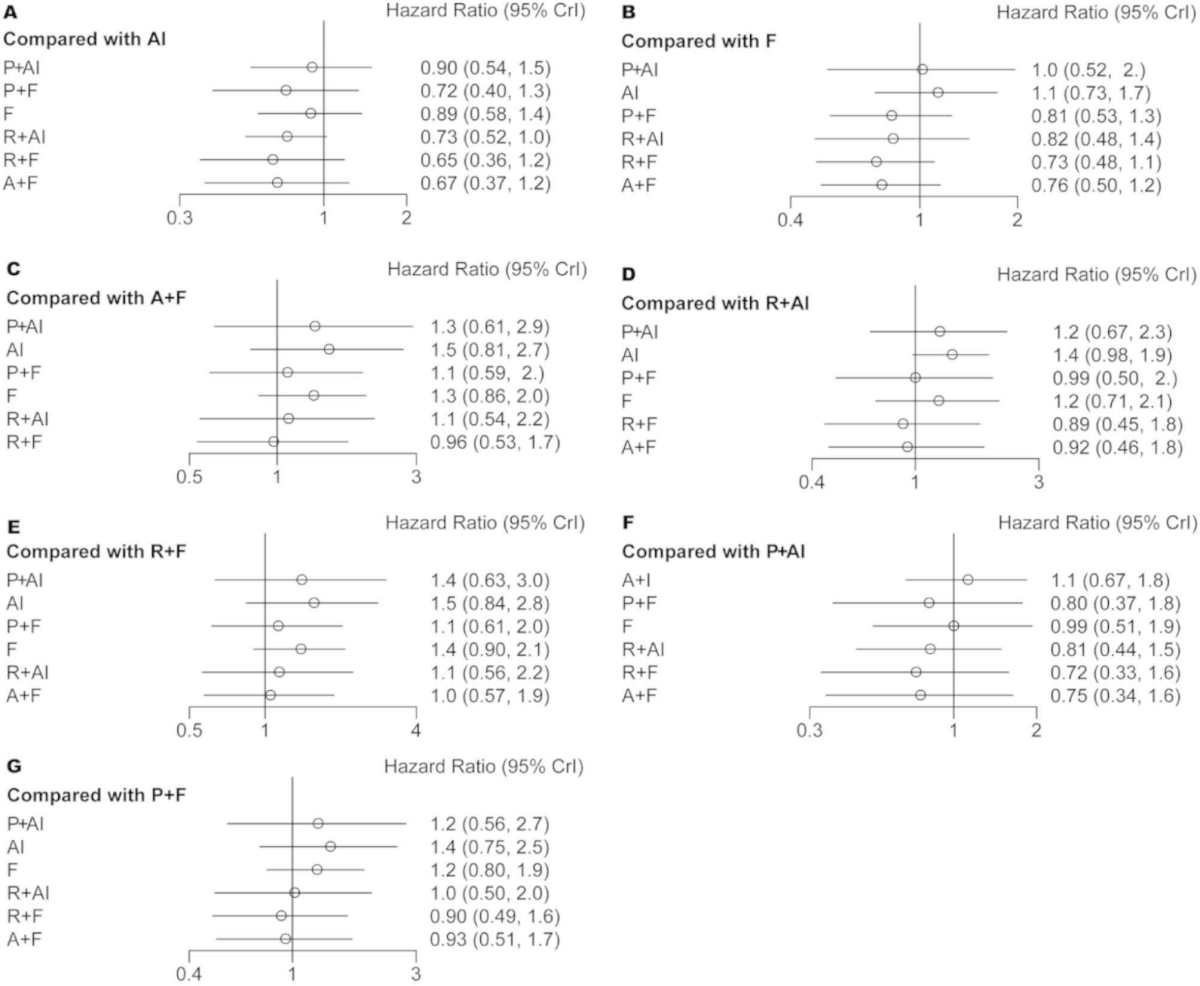
Forest plot of the hazard ratios for OS based on different pairwise comparisons

Among the 9 included studies, 6 studies [41–43, 46, 47] involving 3421 patients reported the HR for OS. The pairwise meta-analysis suggested that the combination of abemaciclib plus fulvestrant (HR = 0.76, 95% CI = 0.61 to 0.94) and ribociclib plus AI (HR = 0.73, 95% CI = 0.58 to 0.91) or fulvestrant (HR = 0.73, 95% CI = 0.59 to 0.90) was associated with improved OS (Supplementary Fig. 2). However, these findings were not supported by the network meta-analysis (abemaciclib plus fulvestrant: HR = 0.76, 95% CI = 0.50 to 1.15; ribociclib plus AI: HR = 0.73, 95% CI = 0.52 to 1.02; ribociclib plus fulvestrant: HR = 0.73, 95% CI = 0.48 to 1.11) (Fig. 5). Similarly, the network meta-analysis indicated no statistical difference between the available treatment combinations of three CDK4/6 inhibitors and different endocrine therapies for OS (Fig. 5).

### Meta-analysis of severe adverse events

All eligible studies [41–49] reported the incidence of severe adverse events. The pairwise meta-analysis revealed that treatment combinations involving three CDK4/6 inhibitors plus AI or fulvestrant were associated with a higher incidence of severe adverse events compared to AI or fulvestrant alone (Supplementary Fig. 3). However, the network meta-analysis confirmed increased incidence only for specific combinations: ribociclib (OR = 9.46, 95% CI = 2.07 to 43.14) or palbociclib (OR = 10.83, 95% CI = 2.36 to 50.93) plus AI and abemaciclib (OR = 4.79, 95% CI = 1.40 to 16.13) or palbociclib (OR = 6.30, 95% CI = 1.03 to 40.68) plus fulvestrant (Supplementary Fig. 4). However, no statistically significant differences were observed between the available treatment combinations of the three CDK4/6 inhibitors and different endocrine therapies in the network meta-analysis (Supplementary Fig. 4).

### Rank Probabilities

**Fig. 6:**
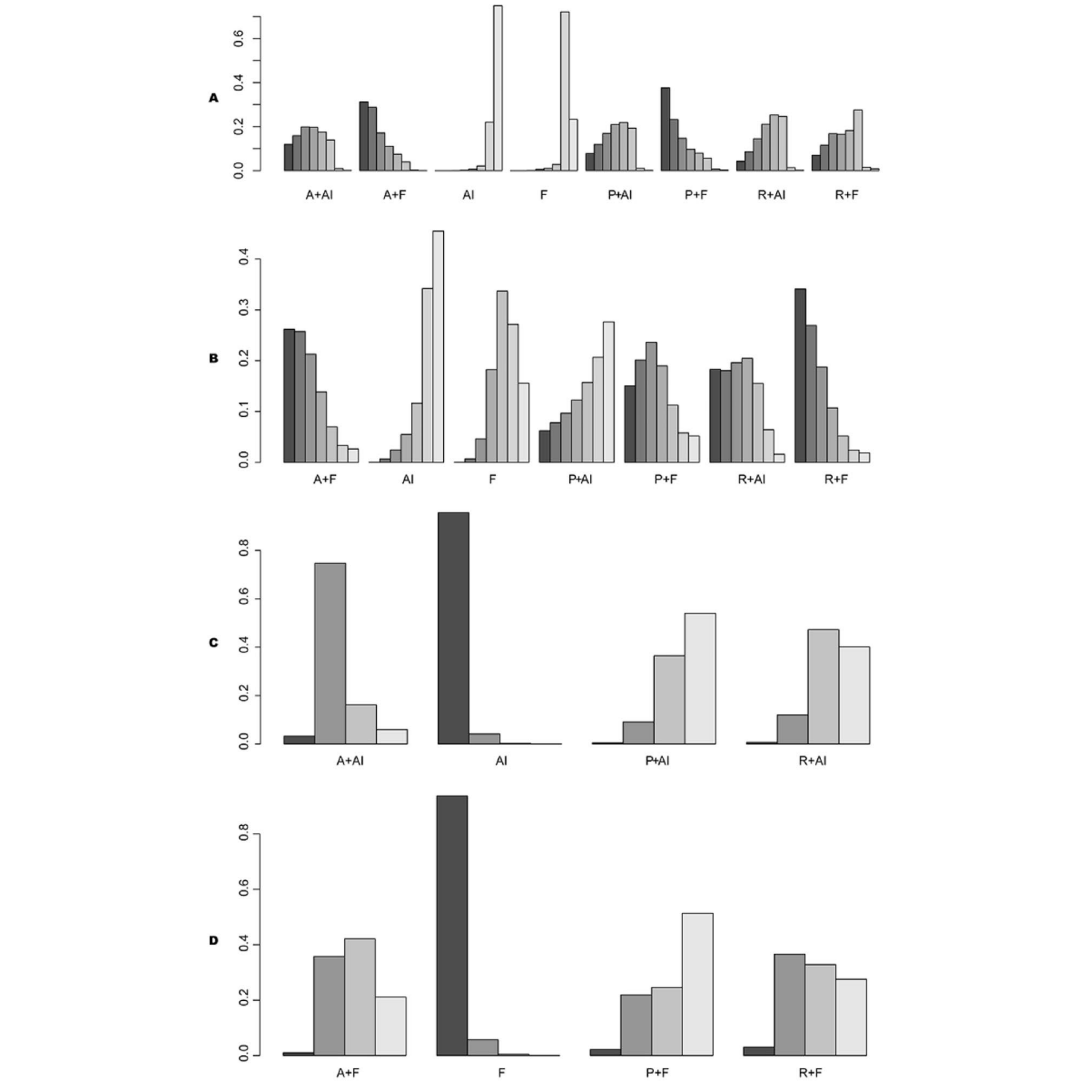
Ranking plot based on the probabilities of interventions in the analysis of secondary outcomes. PFS **(A)**, OS **(B)**, and severe AEs based on different endocrine therapies including AI **(C)** and fulvestrant **(D)**. Treatments are ranked according to their chance of being the best treatment, and the height of each column reflects the probability of the rank

The rankings of all available treatment combinations are presented in Fig. 6. Regarding PFS, palbociclib plus fulvestrant had the highest likelihood of being the most effective regimen (SUCRA = 37.65%), followed by abemaciclib plus fulvestrant (SUCRA = 28.76%) (Fig. 6a). For OS, ribociclib plus fulvestrant was identified as the most effective regimen (SUCRA = 34.11%), with abemaciclib plus fulvestrant ranking second (SUCRA = 25.75%) (Fig. 6b). In terms of severe adverse events, the least desirable regimens were palbociclib plus AI (SUCRA = 53.98%) (Fig. 6c) and palbociclib plus fulvestrant (SUCRA = 51.37%) (Fig. 6d).

### Convergence Assessment

To assess the convergence of the Markov Chain Monte Carlo (MCMC) simulation in our network meta-analysis, we calculated the potential scale reduction factor (PSRF) value. The PSRF value was close to 1, indicating satisfactory convergence of the MCMC simulation.

## Discussion

Although endocrine therapy is recommended for the treatment of metastatic or advanced BC patients with HR+/HER2- as the first-line treatment unless a visceral crisis or life-threatening situation [8, 9], some patients still suffer from recurrence and or suffer from progress due to primary [10] or acquired drugs resistance [11]. Considering the association CDK4/6 with endocrine therapy resistance [13], treatment combinations of different CDK4/6 inhibitors and endocrine therapy are popularly investigated [15]. It’s exciting that several meta-analyses [16–21] confirmed the efficacy and safety of CDK4/6 inhibitors combined with endocrine therapy among metastatic or advanced BC with HR+/HER2-. Unfortunately, it remains unclear which combinations of three CDK4/6 inhibitors with two endocrine therapies maybe the optimal option for treating metastatic or advanced BC with HR+/HER2-. In this network meta-analysis, we introduced results from a published meta-analysis [50] to explore the comparative efficacy and safety of all available treatment combinations of three CDK4/6 inhibitors and two endocrine therapies for the PFS and OS. Our results confirmed the efficacy of various treatment combinations of three CDK4/6 inhibitors and two endocrine therapies in prolonging PFS compared with endocrine therapy alone, and palbociclib plus fulvestrant and abemaciclib plus fulvestrant were likely the most effective regime. No statistical differences were detected between various combinations. Meanwhile, ribociclib plus fulvestrant and abemaciclib plus fulvestrant were likely the most effective regime, although all available treatment combinations, including CDK4/6 inhibitors combined with different endocrine therapies and individual endocrine therapy, were comparable regarding OS. In addition, our network meta-analysis also suggested that palbociclib plus AI or fulvestrant, ribociclib plus AI, and abemaciclib plus fulvestrant significantly increased the incidence of severe AEs compared with endocrine therapy alone, and palbociclib plus AI and palbociclib plus fulvestrant were the worst combination in terms of therapeutic safety. Also, no statistical differences were detected between combinations of three CDK4/6 inhibitors and two endocrine therapies. Six pairwise meta-analyses [16–21] have investigated the comparative efficacy and safety of CDK4/6 inhibitors combined with endocrine therapy and endocrine therapy alone. However, only two meta-analyses [18, 21] designed subgroup analyses to investigate the comparative efficacy and safety of combining three CDK4/6 inhibitors with endocrine therapy and endocrine therapy alone. Meta-analysis performed by Lin et al. [18] suggested that whether the combination of CDK4/6 with AI or fulvestrant or the combination of ribociclib or abemaciclib with endocrine therapy significantly prolonged OS compared with endocrine therapy alone based on six eligible studies. Unfortunately, this meta-analysis did not differentiate the differences in the combination of each CDK4/6 inhibitor plus each endocrine therapy, leading to inconsistent results with our network meta-analysis. Furthermore, our meta-analysis included additional 3 studies to generate more reliable results. In addition, a meta-analysis performed by Zheng and colleagues also used a similar method to differentiate the efficacy and safety of various combinations of three CDK4/6 inhibitors with endocrine therapy [21], revealing that combinations of three CDK4/6 inhibitors with endocrine therapy were significantly associated with the decreased PFS and the increased incidence of severe AEs, and ribociclib plus endocrine therapy was related to longer OS. It’s noted that AI and fulvestrant inhibit estrogen production by regulating different pathways, therefore exerting different efficacy, as found in a previous meta-analysis [50]. Therefore, it’s more reasonable to separately differentiate various combinations of different CDK4/6 inhibitors with two endocrine therapies. This viewpoint can be further supported by the fact that combinations of three CDK4/6 inhibitors with two endocrine therapies were associated with different risks of severe AEs. Three network meta-analyses focused on a similar topic have also been published recently [22–24]; however, no one differentiated the types of CDK4/6 inhibitors [22]. Desnoyers and colleagues performed a network meta-analysis to majorly investigate the safety of different CDK4/6 inhibitors in metastatic BC [23] and suggested no statistical difference in comparison of ribociclib versus palbociclib and comparison of abemaciclib versus palbociclib regarding PFS and OS after differentiating endocrine therapies to be AI and fulvestrant, which were consistent with our findings. In addition, this meta-analysis also investigated the risk of three CDK4/6 inhibitors plus different endocrine therapies on the occurrence of specific AEs and suggested that ribociclib and abemaciclib were associated with lower severe neutropenia but higher gastrointestinal toxicity compared with palbociclib. However, no statistical difference was detected in our meta-analysis due to severe AEs being regarded as an individual outcome. Another meta-analysis investigated the efficacy and safety of CDK4/6 and PI3K/AKT/mTOR inhibitors as second-line treatment in postmenopausal patients with HR+/HER2- metastatic BC [24] and revealed no statistical differences between three combinations of three CDK4/6 inhibitors with fulvestrant regarding PFS and OS, which was consistent with our findings. This meta-analysis also showed a benefit for ribociclib plus fulvestrant in terms of severe AEs compared to abemaciclib plus fulvestrant and palboclclib plus fulvestrant, which was inconsistent with our results. It’s noted that this study only considered the combined risk of severe neutropenia, leukopenia, and anemia; however, our network meta-analysis estimated the combined risk of all reported severe AEs. Certainly, it’s essential to investigate the risk of various combinations of severe hematologic AEs under the inspiration of these positive findings [24].

Previous results showed that endocrine therapies were commonly used to treat hormone receptor-positive cancer, but cancer cells may develop resistance to these therapies over time. CDK4/6 inhibitors targeted the cell cycle, preventing cancer cells from progressing through the cell division process. The results of RCTs and this meta-analysis showed better effectiveness when combining these two kinds of therapies, which indicated synergistic action between these two therapies. However, different combinations of these two kinds of therapies showed different effectiveness, especially using different outcomes to compare them. Therefore, the next step is to study pharmacological mechanisms of different therapies and find an optimal combination to treat breast cancer. Our network meta-analysis has several methodological strengths as follows: (a) we applied asystematic literature retrieval strategy to reduce the risk of publication bias; (b) we applied ranking probability to distinguish subtle differences among all available treatment combinations of CDK4/6 inhibitors with different endocrine therapies; (c) our network meta-analysis was the first comparison of direct and indirect regimes, which incorporated all available data to investigate the comparative efficacy and safety of all available treatment combinations more precisely; (d) the good convergence of MCMC simulation ensure the accuracy of the estimates; and (e) all included studies were RCTs, so the evidence was of high quality. Nevertheless, our network meta-analysis has also some limitations as follows: (a) we did not identify direct comparison of different combinations of CDK4/6 inhibitors plus endocrine therapies, therefore causing estimates to be inaccurate; (b) we did not introduce subgroup analysis to investigate the impact of treatment strategies (first-, second-, or subsequent-line), clinical characteristics of patients (premenopausal status, postmenopausal status, and any menopausal status), and doses of CDK4/6 inhibitors on the pooled results due to insufficient number of eligible studies; (c) our network meta-analysis included more eligible studies compared with previous meta-analyses; however, more comparisons were constructed based on limited eligible studies, which inevitably leads to an underestimation of the validity of the analysis; and (d) our literature search was limited to studies published in English, which may lead to language bias and have a negative impact on the validity of pooled results.

## Conclusions

In conclusion, based on our network meta-analysis, the combinations of abemaciclib plus fulvestrant or ribociclib plus AI appear to be promising options for the treatment of HR+/HER2- metastatic or advanced breast cancer. These combinations demonstrate superior efficacy and safety compared to other available treatment options. However, further randomized controlled trials (RCTs) are necessary to provide more robust evidence and compare the efficacy and safety of different treatment combinations involving three CDK4/6 inhibitors and two endocrine therapies.

### Electronic supplementary material

Below is the link to the electronic supplementary material.

Supplementary Material 1

## Data Availability

Full datasets generated during and/or analyzed during the current study are available from the corresponding author on reasonable request. Additional data are available in the supplementary materials.

## List of abbreviations

ABC: Advanced breast cancer
ETs: Endocrine therapies
PFS: Progression-free survival
AI: Aromatase inhibitor
OS: Overall survival
AEs: Adverse events
BC: Breast cancer
FDA: Food and Drug Administration
PSRF: Potential scale reduced factor

## References

[1] Sung H, Ferlay J, Siegel RL, Laversanne M, Soerjomataram I, Jemal A (2021). Global Cancer Statistics 2020: GLOBOCAN estimates of incidence and Mortality Worldwide for 36 cancers in 185 countries. CA Cancer J Clin.

[2] Cardoso F, Spence D, Mertz S, Corneliussen-James D, Sabelko K, Gralow J (2018). Global analysis of advanced/metastatic breast cancer: Decade report (2005–2015). Breast.

[3] Cardoso F, Harbeck N, Fallowfield L, Kyriakides S, Senkus E (2012). Locally recurrent or metastatic breast cancer: ESMO Clinical Practice Guidelines for diagnosis, treatment and follow-up. Ann Oncol.

[4] Perou CM, Sørlie T, Eisen MB, van de Rijn M, Jeffrey SS, Rees CA (2000). Molecular portraits of human breast tumours. Nature.

[5] Cancer Genome Atlas Network (2012). Comprehensive molecular portraits of human breast tumours. Nature.

[6] Howlader N, Cronin KA, Kurian AW, Andridge R (2018). Differences in breast Cancer survival by Molecular Subtypes in the United States. Cancer Epidemiol Biomarkers Prev.

[7] Ginsburg O, Bray F, Coleman MP, Vanderpuye V, Eniu A, Kotha SR (2017). The global burden of women’s cancers: a grand challenge in global health. Lancet.

[8] Rugo HS, Rumble RB, Macrae E, Barton DL, Connolly HK, Dickler MN (2016). Endocrine therapy for hormone receptor-positive metastatic breast Cancer: American Society of Clinical Oncology Guideline. J Clin Oncol.

[9] Cardoso F, Senkus E, Costa A, Papadopoulos E, Aapro M, André F (2018). 4th ESO-ESMO International Consensus Guidelines for advanced breast Cancer (ABC 4)†. Ann Oncol.

[10] Milani A, Geuna E, Mittica G, Valabrega G (2014). Overcoming endocrine resistance in metastatic breast cancer: current evidence and future directions. World J Clin Oncol.

[11] Sestak I, Dowsett M, Zabaglo L, Lopez-Knowles E, Ferree S, Cowens JW (2013). Factors predicting late recurrence for estrogen receptor-positive breast cancer. J Natl Cancer Inst.

[12] Spring L, Bardia A, Modi S (2016). Targeting the cyclin D-cyclin-dependent kinase (CDK) 4/6-retinoblastoma pathway with selective CDK 4/6 inhibitors in hormone receptor-positive breast cancer: rationale, current status, and future directions. Discov Med.

[13] Thangavel C, Dean JL, Ertel A, Knudsen KE, Aldaz CM, Witkiewicz AK (2011). Therapeutically activating RB: reestablishing cell cycle control in endocrine therapy-resistant breast cancer. Endocr Relat Cancer.

[14] Goetz MP, Gradishar WJ, Anderson BO, Abraham J, Aft R, Allison KH (2019). NCCN Guidelines Insights: breast Cancer, Version 3.2018. J Natl Compr Canc Netw.

[15] Turner NC, Neven P, Loibl S, Andre F (2017). Advances in the treatment of advanced oestrogen-receptor-positive breast cancer. Lancet.

[16] Li J, Fu F, Yu L, Huang M, Lin Y, Mei Q (2020). Cyclin-dependent kinase 4 and 6 inhibitors in hormone receptor-positive, human epidermal growth factor receptor-2 negative advanced breast cancer: a meta-analysis of randomized clinical trials. Breast Cancer Res Treat.

[17] Li Y, Li L, Du Q, Li Y, Yang H, Li Q (2021). Efficacy and safety of CDK4/6 inhibitors combined with endocrine therapy in HR+/HER-2- ABC Patients: a systematic review and Meta-analysis. Cancer Invest.

[18] Lin M, Chen Y, Jin Y, Hu X, Zhang J (2020). Comparative overall survival of CDK4/6 inhibitors plus endocrine therapy vs. endocrine therapy alone for hormone receptor-positive, HER2-negative metastatic breast cancer. J Cancer.

[19] Ramos-Esquivel A, Hernández-Romero G, Landaverde DU (2020). Cyclin–dependent kinase 4/6 inhibitors in combination with fulvestrant for previously treated metastatic hormone receptor–positive breast cancer patients: a systematic review and meta–analysis of randomized clinical trials. Cancer Treat Res Commun.

[20] Xu ZH, Zhang H, Wei DH, Xie LL, Xu CS (2020). Cyclin-dependent kinase 4/6 inhibitor in combination with endocrine therapy versus endocrine therapy only for advanced breast cancer: a systematic review and meta-analysis. Translational Cancer Research.

[21] Zheng J, Wu J, Wang C, Zhuang S, Chen J, Ye F (2020). Combination cyclin-dependent kinase 4/6 inhibitors and endocrine therapy versus endocrine monotherapy for hormonal receptor-positive, human epidermal growth factor receptor 2-negative advanced breast cancer: a systematic review and meta-analysis. PLoS ONE.

[22] Brandão M, Maurer C, Ziegelmann PK, Pondé NF, Ferreira A, Martel S et al. Endocrine therapy-based treatments in hormone receptor-positive/HER2-negative advanced breast cancer: systematic review and network meta-analysis. ESMO Open. 2020;5(4). 10.1136/esmoopen-2020-000842 PMC7451473 32847835

[23] Desnoyers A, Nadler MB, Kumar V, Saleh R, Amir E (2020). Comparison of treatment-related adverse events of different cyclin-dependent kinase 4/6 inhibitors in metastatic breast cancer: a network meta-analysis. Cancer Treat Rev.

[24] Leung JH, Leung HWC, Wang SY, Huang SS, Chan ALF (2021). Efficacy and safety of CDK4/6 and PI3K/AKT/mTOR inhibitors as second-line treatment in postmenopausal patients with hormone receptor-positive, HER-2-negative metastatic breast cancer: a network meta-analysis. Expert Opin Drug Saf.

[25] Mbuagbaw L, Rochwerg B, Jaeschke R, Heels-Andsell D, Alhazzani W, Thabane L (2017). Approaches to interpreting and choosing the best treatments in network meta-analyses. Syst Rev.

[26] Page MJ, Moher D, Bossuyt PM, Boutron I, Hoffmann TC, Mulrow CD (2021). PRISMA 2020 explanation and elaboration: updated guidance and exemplars for reporting systematic reviews. BMJ.

[27] Hutton B, Salanti G, Caldwell DM, Chaimani A, Schmid CH, Cameron C (2015). The PRISMA extension statement for reporting of systematic reviews incorporating network meta-analyses of health care interventions: checklist and explanations. Ann Intern Med.

[28] Higgins JPT, Green S. Cochrane Handbook for Systematic Reviews of Interventions Version 5.1.0 [updated March 2011]. The Cochrane Collaboration, 2011 Available from wwwhandbookcochraneorg. 2011.

[29] Higgins JP, Altman DG, Gøtzsche PC, Jüni P, Moher D, Oxman AD (2011). The Cochrane collaboration’s tool for assessing risk of bias in randomised trials. BMJ.

[30] Caldwell DM, Ades AE, Higgins JP (2005). Simultaneous comparison of multiple treatments: combining direct and indirect evidence. BMJ.

[31] Dias S, Welton NJ, Caldwell DM, Ades AE (2010). Checking consistency in mixed treatment comparison meta-analysis. Stat Med.

[32] Lumley T (2002). Network meta-analysis for indirect treatment comparisons. Stat Med.

[33] Palma Pérez S, Delgado RM (2006). [Practical considerations on detection of publication bias]. Gac Sanit.

[34] Cipriani A, Higgins JP, Geddes JR, Salanti G (2013). Conceptual and technical challenges in network meta-analysis. Ann Intern Med.

[35] Thom H, White IR, Welton NJ, Lu G (2019). Automated methods to test connectedness and quantify indirectness of evidence in network meta-analysis. Res Synth Methods.

[36] Stephen BROOKS, GELMAN P, Andrew (1998). General methods for monitoring convergence of iterative simulations. J Comput Graphical Stat.

[37] Burger DA, Schall R (2015). A bayesian nonlinear Mixed-Effects Regression Model for the characterization of early bactericidal activity of tuberculosis drugs. J Biopharm Stat.

[38] Veroniki AA, Vasiliadis HS, Higgins JP, Salanti G (2013). Evaluation of inconsistency in networks of interventions. Int J Epidemiol.

[39] Higgins JP, Thompson SG (2002). Quantifying heterogeneity in a meta-analysis. Stat Med.

[40] Albert I, Makowski D (2019). Ranking crop species using mixed treatment comparisons. Res Synth Methods.

[41] Hortobagyi GN, Stemmer SM, Burris HA, Yap YS, Sonke GS, Paluch-Shimon S (2018). Updated results from MONALEESA-2, a phase III trial of first-line ribociclib plus letrozole versus placebo plus letrozole in hormone receptor-positive, HER2-negative advanced breast cancer. Ann Oncol.

[42] Turner NC, Slamon DJ, Ro J, Bondarenko I, Im SA, Masuda N (2018). Overall survival with palbociclib and fulvestrant in advanced breast Cancer. N Engl J Med.

[43] Im SA, Lu YS, Bardia A, Harbeck N, Colleoni M, Franke F (2019). Overall survival with Ribociclib plus endocrine therapy in breast Cancer. N Engl J Med.

[44] Johnston S, Martin M, Di Leo A, Im SA, Awada A, Forrester T (2019). MONARCH 3 final PFS: a randomized study of abemaciclib as initial therapy for advanced breast cancer. NPJ Breast Cancer.

[45] Rugo HS, Finn RS, Diéras V, Ettl J, Lipatov O, Joy AA (2019). Palbociclib plus letrozole as first-line therapy in estrogen receptor-positive/human epidermal growth factor receptor 2-negative advanced breast cancer with extended follow-up. Breast Cancer Res Treat.

[46] Finn RS, Boer K, Bondarenko I, Patel R, Pinter T, Schmidt M (2020). Overall survival results from the randomized phase 2 study of palbociclib in combination with letrozole versus letrozole alone for first-line treatment of ER+/HER2- advanced breast cancer (PALOMA-1, TRIO-18). Breast Cancer Res Treat.

[47] Sledge GW, Toi M, Neven P, Sohn J, Inoue K, Pivot X (2020). The Effect of Abemaciclib Plus Fulvestrant on overall survival in hormone Receptor-Positive, ERBB2-Negative breast Cancer that progressed on endocrine Therapy-MONARCH 2: a Randomized Clinical Trial. JAMA Oncol.

[48] Zhang QY, Sun T, Yin YM, Li HP, Yan M, Tong ZS (2020). MONARCH plus: abemaciclib plus endocrine therapy in women with HR+/HER2- advanced breast cancer: the multinational randomized phase III study. Ther Adv Med Oncol.

[49] Slamon DJ, Neven P, Chia S, Jerusalem G, De Laurentiis M, Im S (2021). Ribociclib plus fulvestrant for postmenopausal women with hormone receptor-positive, human epidermal growth factor receptor 2-negative advanced breast cancer in the phase III randomized MONALEESA-3 trial: updated overall survival. Ann Oncol.

[50] Wang J, Xu B, Wang W, Zhai X, Chen X (2018). Efficacy and safety of fulvestrant in postmenopausal patients with hormone receptor-positive advanced breast cancer: a systematic literature review and meta-analysis. Breast Cancer Res Treat.

