# Supplementary material for: Comparative efficacy and safety of different combinations of three CDK4/6 inhibitors with endocrine therapies in HR+/HER-2 − metastatic or advanced breast cancer patients: a network meta-analysis
Source: BMC Cancer. 2023 Aug 31;23:816. doi: 10.1186/s12885-023-11322-2 (PMC10469949; doi:10.1186/s12885-023-11322-2)
Supplement: Supplementary file 1 — Supplementary Material 1 [file 12885_2023_11322_MOESM1_ESM.docx]

Supplementary Material

## Supplementary Figures


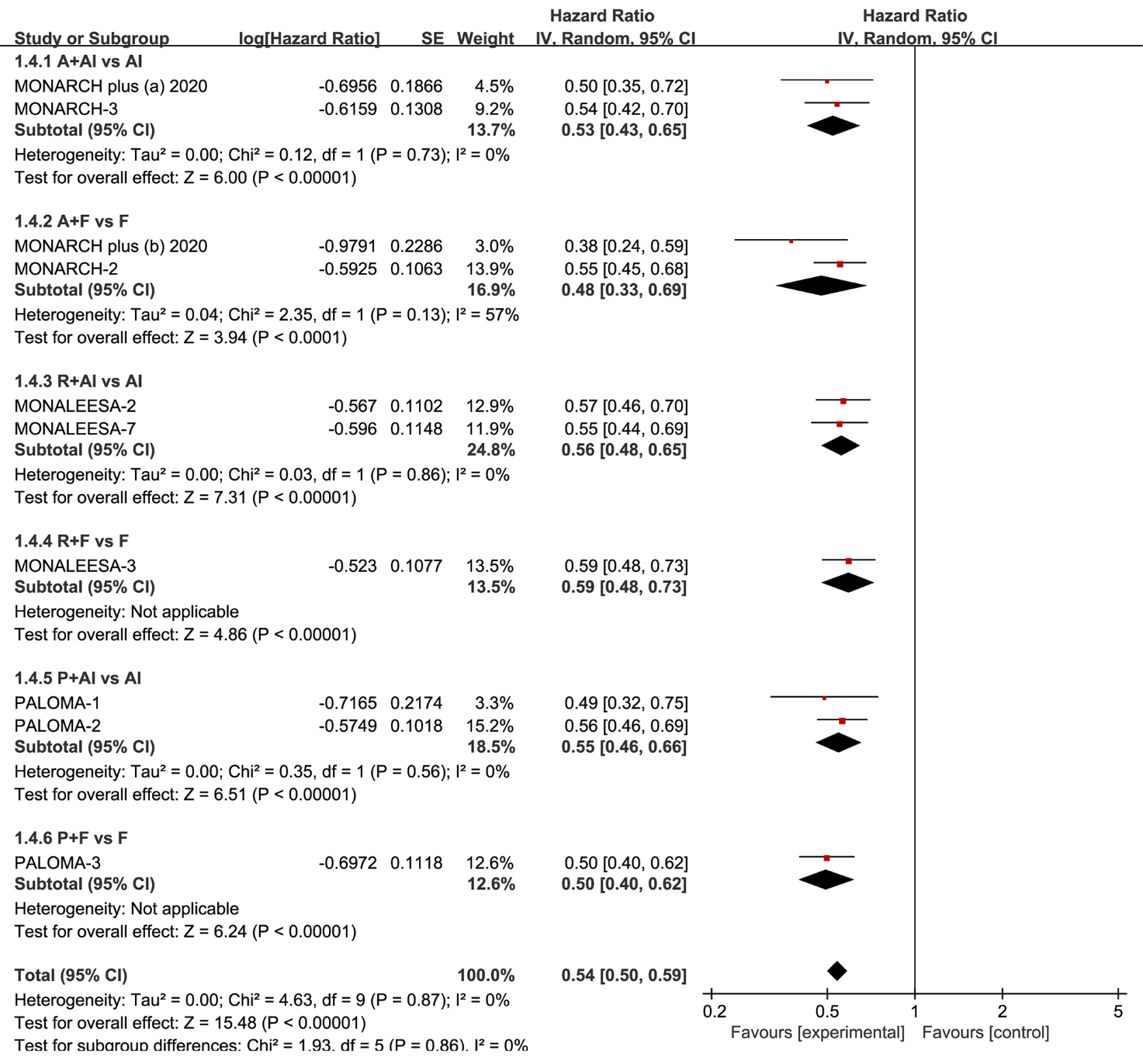


**Figure S1.** Pairwise meta-analysis of the hazard ratios for PFS based on different pairwise comparisons.


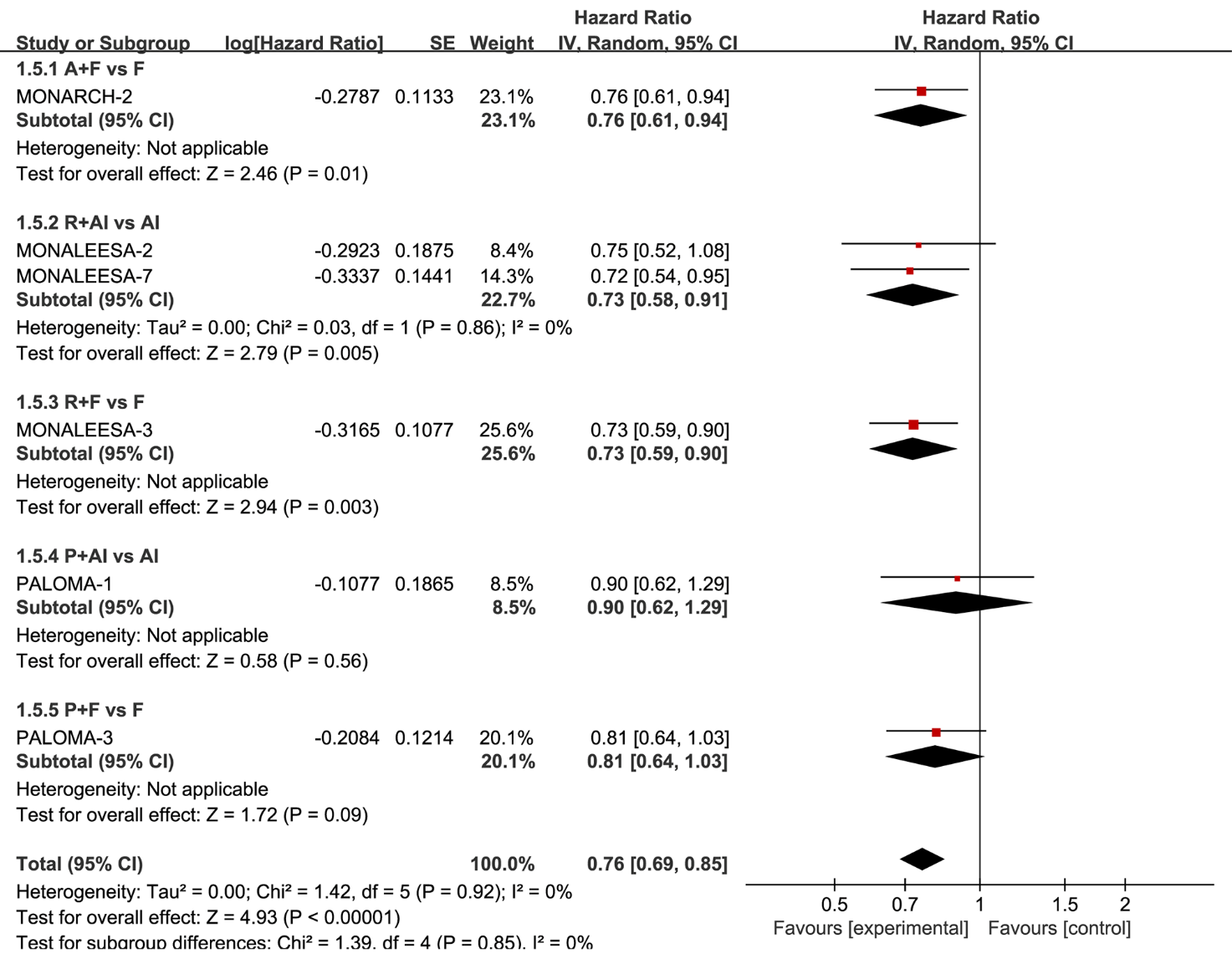


**Figure S2.** Pairwise meta-analysis of the hazard ratios for OS based on different pairwise comparisons.


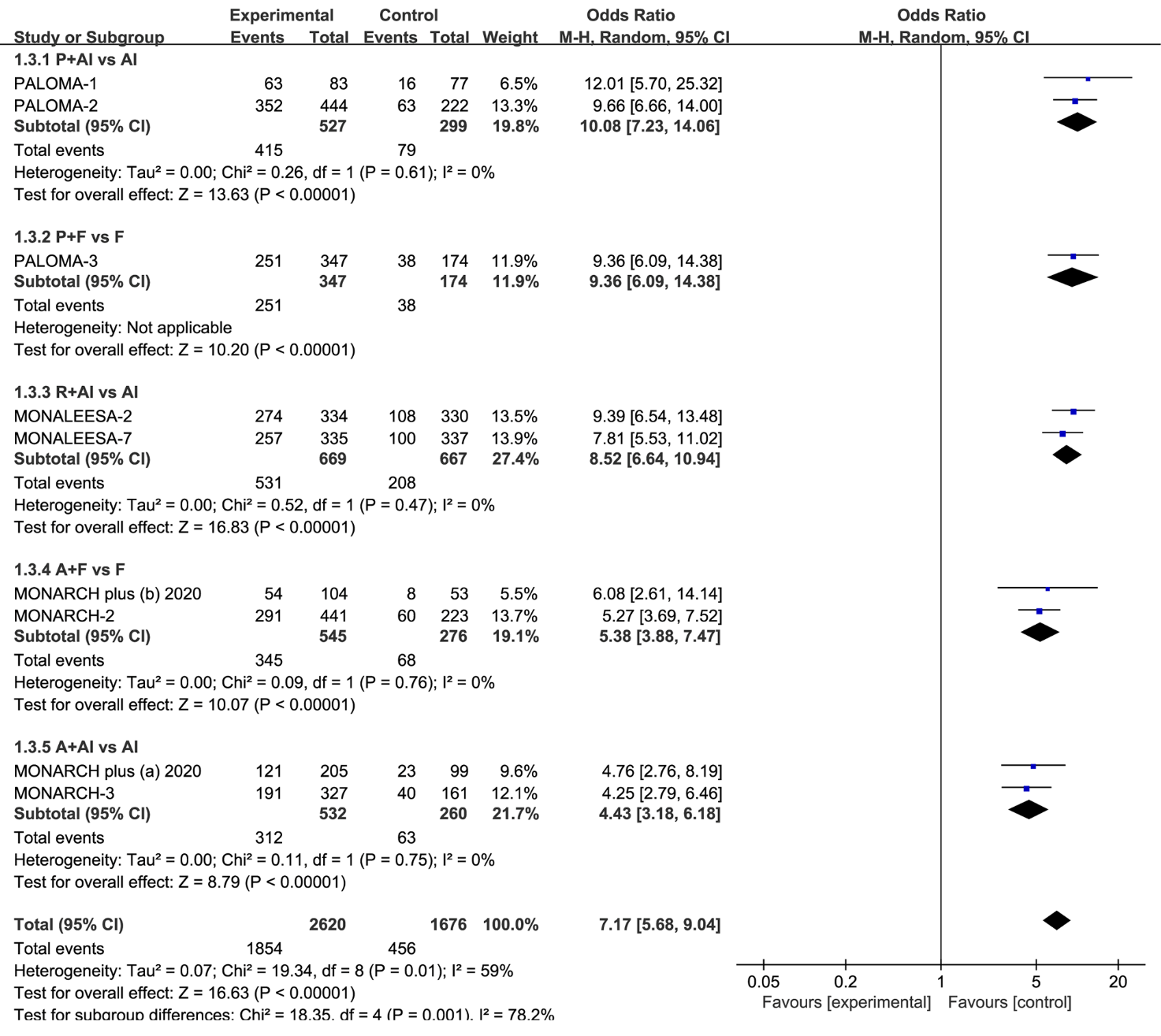


**Figure S3.** Pairwise meta-analysis of the odds ratios for severe AEs based on different pairwise comparisons.


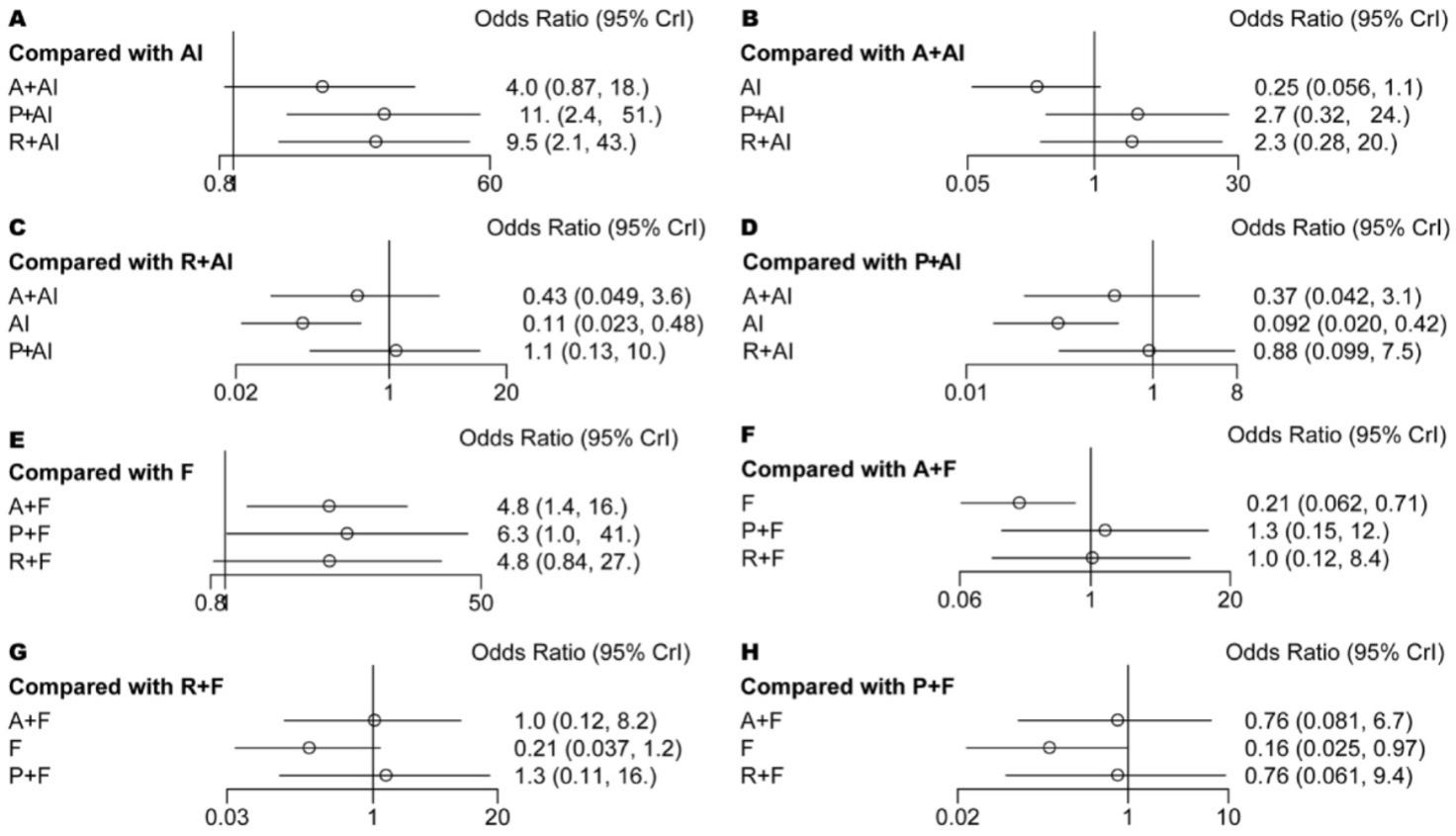


**Supplementary Figure 4.** Forest plot of the odds ratios for severe AEs based on different pairwise comparisons.

## Supplementary Table 1 The search strategies for different databases.

**Pubmed**

| No. | Query | Results |  |
| --- | --- | --- | --- |
| 18 | #17; Filters: Humans, from 2020 - 2021 | 64 |  |
| 17 | #16; Filters: Humans | 167 |  |
| 16 | #5 and #8 and #15 | 250 |  |
| 15 | #10 or #13 or #14 | ######## |  |
| 14 | ((placebos[Title/Abstract]) OR (placebo*[Title/Abstract])) OR (random*[Title/Abstract]) | ######## |  |
| 13 | #11 and #12 | 207,862 |  |
| 12 | (mask*[Title/Abstract]) OR (blind*[Title/Abstract]) | 405,542 |  |
| 11 | (((singl*[Title/Abstract]) OR (doubl*[Title/Abstract])) OR (trebl*[Title/Abstract])) OR (tripl*[Title/Abstract]) | ######## |  |
| 10 | clinical trial[Title/Abstract] | 168,777 |  |
| 9 | ((((((("Randomized Controlled Trial" [Publication Type]) OR "Controlled Clinical Trial" [Publication Type]) OR "Randomized Controlled Trials as Topic"[Mesh]) OR "Random Allocation"[Mesh]) OR "Double-Blind Method"[Mesh]) OR "Single-Blind Method"[Mesh]) OR "Clinical Trial" [Publication Type]) OR "Clinical Trials as Topic"[Mesh] | ######## |  |
| 8 | #6 or #7 | 6,421 |  |
| 7 | ((palbociclib[Title/Abstract]) OR (ribociclib[Title/Abstract])) OR (abemaciclib[Title/Abstract]) | 1,489 |  |
| 6 | (((((cyclin-dependent kinase 4[Title/Abstract] AND 6 inhibitor*[Title/Abstract]) OR (cyclin-dependent kinase 4/6 inhibitor*[Title/Abstract])) OR (CDK4[Title/Abstract] AND CDK6 inhibitor*[Title/Abstract])) OR (CDK4/6 inhibitor*[Title/Abstract])) OR (CDK inhibitor*[Title/Abstract])) OR (CDKi[Title/Abstract]) | 5,771 |  |
| 5 | #3 and #4 | 92,818 |  |
| 4 | (((metastatic[Title/Abstract]) OR (advanced[Title/Abstract])) OR (metastases[Title/Abstract])) OR (metastasis[Title/Abstract]) | 949,304 |  |
| 3 | #2 or #3 | 422,320 |  |
| 2 | (((((((Breast Neoplasm*[Title/Abstract]) OR (Breast Tumor*[Title/Abstract])) OR (Breast Cancer*[Title/Abstract])) OR (Breast Carcinoma*[Title/Abstract])) OR (Mammary Neoplasm*[Title/Abstract])) OR (Mammary Tumor*[Title/Abstract])) OR (Mammary Cancer*[Title/Abstract])) OR (Mammary Carcinoma*[Title/Abstract]) | 347,016 |  |
| 1 | "Breast Neoplasms"[Mesh] | 313,185 |  |
| **Embase** <1974 to 2021 October 12> | | | |
| 1 (Breast Neoplasm* or Breast Tumor* or Breast Cancer* or Breast Carcinoma* or Mammary Neoplasm* or Mammary Tumor* or Mammary Cancer* or Mammary Carcinoma*).af. 650296 | | | |
| 2 exp breast tumor/ 579170 | | | |
| 3 1 or 2 661145 | | | |
| 4 (metastatic or advanced or metastases or metastasis).af. 1861552 | | | |
| 5 3 and 4 191472 | | | |
| 6 ((cyclin-dependent kinase 4 and 6 inhibitor*) or (CDK4 and CDK6 inhibitor*) or cyclin-dependent kinase 4 6 inhibitor* or CDK4 6 inhibitor* or CDK inhibitor* or CDKi).af. 8825 | | | |
| 7 (palbociclib or ribociclib or abemaciclib).af. 5999 | | | |
| 8 6 or 7 12337 | | | |
| 9 (singl* or doubl* or trebl* or tripl*).af. 3424531 | | | |
| 10 (mask* or blind*).af. 689568 | | | |
| 11 9 and 10 372813 | | | |
| 12 (clinical trial or placebos or placebo* or random*).af. 3058772 | | | |
| 13 11 or 12 3111684 | | | |
| 14 exp randomized controlled trial/ 680767 | | | |
| 15 exp controlled clinical trial/ 869138 | | | |
| 16 exp "randomized controlled trial (topic)"/ 212695 | | | |
| 17 exp randomization/ 92276 | | | |
| 18 exp double blind procedure/ 188630 | | | |
| 19 exp single blind procedure/ 44004 | | | |
| 20 exp clinical trial/ 1637808 | | | |
| 21 exp "clinical trial (topic)"/ 370346 | | | |
| 22 13 or 14 or 15 or 16 or 17 or 18 or 19 or 20 or 21 3233780 | | | |
| 23 5 and 8 and 22 1961 | | | |
| 24 limit 23 to (human and embase and yr="2020 -Current") 304 | | | |

**Supplementary Table 2. Basic characteristics of eligible studies.**

| Study | Clinicaltrial ID | Author | Year | Phase | Line | Sample size | Median age, years | Condition | Regimes | | Dose | Median follow-up, months |
| --- | --- | --- | --- | --- | --- | --- | --- | --- | --- | --- | --- | --- |
|  |  |  |  |  |  |  |  |  | Study | Control |  |  |
| PALOMA-1 | NCT00721409 | Finn RS | 2015/2020 | II | First-line | 165 (84 vs 81) | 63 vs64 | Postmenopausal status | P+AI | AI | Palbociclib 125mg daily, 3 weeks on/ 1 week off; letrozole 2.5mg qd | >29.6 vs >27.9 |
| PALOMA-2 | NCT01740427 | Rugo HS | 2016/2019 | II | First-line | 666 (444 vs 222) | 62 vs 61 | Postmenopausal status | P+AI | AI | Palbociclib 125mg daily, 3 weeks on/ 1 week off; letrozole 2.5mg qd | 37.6 vs 37.3 |
| PALOMA-3 | NCT01942135 | Turner NC | 2016/2018 | III | Second- and subsequent line | 521 (347 vs 174) | 57 vs 56 | Any menopausal status | P+F | F | Palbociclib 125mg daily 3 weeks on/1 week off; fulvestrant 500mg q4w | 44.8 |
| MONALEESA-2 | NCT01958021 | Hortobagyi GN | 2016/2018 | III | First-line | 668 (334 vs 334) | 62 vs 63 | Postmenopausal status | R+AI | AI | Ribociclib 600mg daily 3 weeks on/1 week off; letrozole 2.5mg qd | 39.4 |
| MONALEESA-3 | NCT02422615 | Slamon DJ | 2018/2021 | III | First-line | 726 (484 vs 242) | 63 vs 63 | Postmenopausal status | R+F | F | Ribociclib 600mg daily 3 weeks on/ 1 week off; fulvestrant 500mg q4w | 56.3 |
| MONALEESA-7 | NCT02278120 | Im SA | 2018/2019 | III | First-line | 672 (335 vs 337) | 43 vs 45 | Per or peri-menopausal status | R+AI | AI | Ribociclib 600mg daily 3 weeks on/1 week off; 20mg qd; tamoxifen 20mg qd or letrozole 2.5mg qd or anastrozole 1mg qd; goserelin 3.6mg q4w | 34.6 |
| MONARCH-2 | NCT02107703 | Sledge GW Jr | 2017/2020 | III | Second-line | 669 (446 vs 223) | 59 vs 62 | Any menopausal status | A+F | F | Abemaciclib 150mg bid; fulvestrant 500mg q4w | 47.7 |
| MONARCH-3 | NCT02246621 | Johnston S | 2017/2019 | III | First-line | 493 (328 vs 165) | 63 vs 63 | Postmenopausal status | A+AI | AI | Abemaciclib 150mg bid; letrozole 2.5mg qd or anastrozole 1mg qd. | 26.7 |
| MONARCH plus (cohort A) | NCT02763566 | Zhang QY | 2020 | III | First-line | 306 (207 vs 99) | n.r. | Postmenopausal status | A+AI | AI | Abemaciclib 150mg bid; letrozole 2.5mg qd or anastrozole 1mg qd | n.r. |
| MONARCH plus (cohort B) | NCT02763566 | Zhang QY | 2020 | III | First- and subsequent-line | 157 (104 vs 53) | n.r. | Postmenopausal status | A+F | F | Abemaciclib 150mg bid; fulvestrant 500mg q4w | n.r. |

P, palbociclib; R, ribociclib; A, abemaciclib; AI, aromatase inhibitor; F, fulvestrant; HR, hazard ratio; PFS, progression-free survival; OS, overall survival; n.r., not reported.
